# Nematodes: The Worm and Its Relatives

**DOI:** 10.1371/journal.pbio.1001050

**Published:** 2011-04-19

**Authors:** Mark Blaxter

**Affiliations:** Institute of Evolutionary Biology, The University of Edinburgh, Ashworth Laboratories, Edinburgh, United Kingdom

Browse recently published articles in most issues of leading journals, and there will be mention of “the worm”. What is this worm, why is it so keenly studied by so many, and what has it told us about the diversity of life? And why this worm, and not one of the many other worms?

## *Caenorhabditis elegans* Is *the* Worm

The worm is *Caenorhabditis elegans*, a small, bacteriovorous nematode (or roundworm) first described by Emile Maupas in 1900 [1]. While *C. elegans* had been known and studied in the laboratories of nematologists for many years, it was not until Sydney Brenner in Cambridge, United Kingdom, selected this species for his new programme in genetic research [2],[3] that it became a global phenomenon. He wanted a species that was easy to keep, that had tractable genetics (so that mutants could be isolated and crosses made), and that was easy to observe. Brenner attracted a remarkable team of geneticists to join him, and *C. elegans* researchers have won three Nobel prizes for discoveries made using his new model organism.

**Figure 1:**
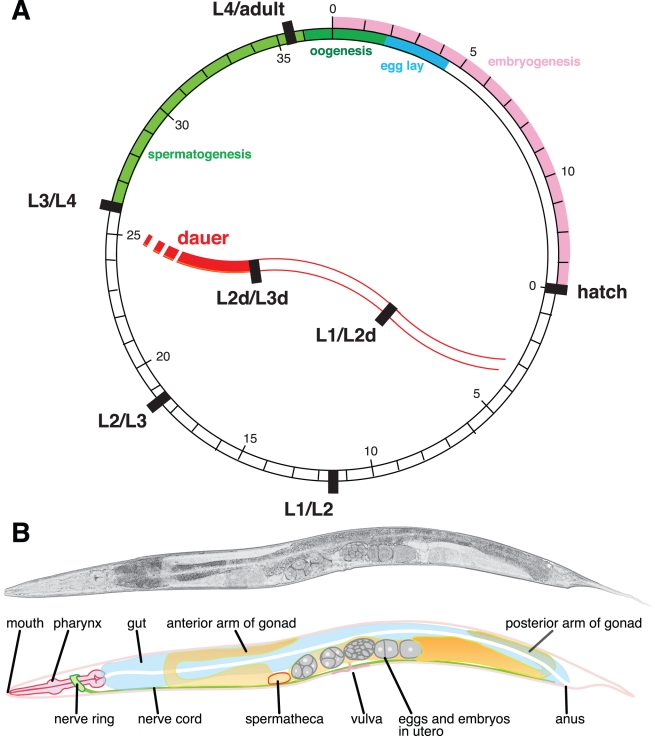
The simple life cycle and anatomy of *C. elegans*. (A) *C. elegans* has a direct life cycle, with eggs developing through four larval stages into sexual adults. The larvae resemble the adults except in the lack of fully developed gonads, and their smaller size. The illustration shows the timing of developmental events at 25°C, with hours since fertilisation on the outside of the circle, and hours since hatching on the inside. Moults are indicated by solid black bars. In the hermaphrodite, the first ∼250 germ cells develop as sperm (after the L3 to L4 moult); later germ cells develop as oocytes. In conditions of overcrowding, starvation, or high temperature, *C. elegans* L1 commit to enter an alternate developmental pathway (via a lipid-storing alternate L2d) that results in the production of a diapausal dauer (“enduring”) L3d. The L3d is non-feeding, resistant to environmental insult, and displays arrested ageing. The L3d resumes development when exposed to sufficient food resources. Other nematodes also have a five-stage life cycle, punctuated by four moults, and many species, including parasites, also have a dauer-like L3 stage. (B) The adult hermaphrodite anatomy is simply observed under light microscopy. Above is an adult animal (length ∼1 mm). In the cartoon below the major organ systems are indicated.

So, why *C. elegans*? One key feature of this nematode is how easy it has turned out to be to grow, observe, analyse, and manipulate (See Box 1). It thrives in simple petri-dish culture, and has a simple life cycle (Figure 1). It is small, but easy to visualise under the microscope. It is see-through at all stages of development, facilitating the analysis of changes in development, or following experimental manipulation. *C. elegans* is an animal, and so has, like other animals, muscles, a nervous system, a digestive system, skin, and so on. Remarkably, and attractively, in *C. elegans* all these organs and tissues are built with very few cells: Brenner's postdoc John Sulston counted 558 nuclei in a hatching larva, and 959 in an adult hermaphrodite (excluding the germline) [4]–[6]. Sulston and colleagues mapped the origins and fates of all these nuclei during development in the beautifully transparent embryos. *C. elegans* embryos undergo a stereotypical pattern of cleavage from the just-fertilised zygote to the emerging first stage larva, such that (with a few important exceptions) the cell lineage is invariant [4]–[6]. For each cell in any embryo, it is possible to say with certainty where it came from (which cells in earlier embryos were its progenitors) and which cells (and tissues) the cell would contribute to the mature animal.

**Box 1.** Setting Up to Study the Worm

There are many small animal species, yet *C. elegans* is the pre-eminent model. This is in part due to the ease of culture, manipulation, and observation of this nematode. Starting a lab to work on the worm requires, initially, only a few key tools: an incubator that maintains a ∼20°C environment, a good dissection microscope, and a good Internet connection. To observe developing embryos, an inverted Nomarski (differential interference contrast) compound microscope is sufficient.

- *C. elegans* does not need complex rearing conditions: it feeds on bacteria, and in the lab can be maintained at room temperature on agar plates covered with a lawn of the standard molecular biology bacterium *Escherichicia coli*. No bio-containment is required.
- It is small (adults are ∼1 mm in length), and thus millions of nematodes can be housed in a small space.
- It is transparent throughout the life cycle, making it easy to directly observe changes at the cellular level using standard live microscopy. This includes following the development of the embryo from fertilisation to hatching.
- It has a short life cycle, taking only 3 days to proceed from a fertilised egg to a sexual adult (Figure 1). Thus, genetic experiments involving multiple generations can be completed in only a few days.
- Propagation is simple, as the standard sexual morph is the self-fertilising hermaphrodite. Because of this mode of reproduction, issues of inbreeding depression (where inbreeding results in lowered reproductive fitness of lines because of homozygous deleterious mutations) are largely absent. Matrilineal stocks can be propagated for decades.
- Genetic crossing is still possible, as *C. elegans* can also exist as fertile males that successfully mate with hermaphrodites to produce outcross offspring.
- *C. elegans* can be cryopreserved at −80°C, allowing strains to be archived securely.
- The *C. elegans* community has sponsored strain and genetic resources collections, and these are searchable online. Mutant strains can be ordered online, and delivered in days through standard mail.
- The genome sequence, and resources of transgenic strains and of RNA interference reagents targeting all the genes in the genome, make the process of identifying and detailing the genetic underpinnings of traits streamlined.

Many successful researchers have started their independent *C. elegans* labs by using these basic resources to perform imaginative screens for mutations affecting particular phenotypes of interest, and thus identifying new genes controlling key biological systems.

*C. elegans* “behaves” much as other animals do—finding food, finding mates, and avoiding danger. However, these behaviours are achieved with a tiny number of neurons: only 302 cell nuclei are present in the adult hermaphrodite nervous system. John White, Sydney Brenner, and colleagues used serial transmission electron microscopy to reconstruct the anatomy and, more importantly, connectivity of this simple nervous system in individual animals [7]. The neurons could be grouped into 118 classes, and their interactions through 7,600 synapses were identified. It remains the only animal nervous system with such a complete wiring diagram, but, frustratingly, it proved impossible to “compute” *C. elegans* behaviour from this, and thus the dynamic field of *C. elegans* neurobiology was founded.

## From Locus to Gene to Genome

Brenner's first paper [3] described 619 visibly mutant strains picked from spontaneously arising variants and from cultures treated with the mutagen ethyl methanesulphonate. These were mapped and used to define six linkage groups, confirming the karyotype (2n = 12) and mode of sex determination (males have 2n = 11, and sex is determined by the number of X chromosomes). Importantly, these mutants include several that affect development, changing or deleting the fates of cells in the lineage. From these small, promising beginnings, a worldwide community of *C. elegans* researchers grew, using mutagenesis and careful developmental and biological analyses to reveal the genetic underpinnings of development, neurosensation, ageing, and many other phenotypes. The *C. elegans* research field has been openly collaborative from the beginning, with *The Worm Breeder's Gazette* an early example of open-access publishing of research findings by and to a self-defined community (see Table 1). One of the key products of this collaboration was the development of a genetic map, placing all the loci identified across the world on a common framework [8].

**Table 1:** Resources for *C. elegans* and other nematodes.

Name	URL	Content
WormBase	http://www.wormbase.org/	The *C. elegans* genome database, including the genome sequence, expression pattern data, and genetic mapping information. Also includes comparative analyses of the genomes of other nematodes.
WormBook	http://www.wormbook.org/	The online, peer-reviewed, open-access textbook on *C. elegans* biology, genetics, development, and evolution. Includes the archive of *Worm Breeder's Gazette*. Freely searchable and downloadable.
WormAtlas	http://www.wormatlas.org/	The online virtual worm. This extraordinarily detailed atlas is built from high resolution electron micrographs and includes gene expression patterns and neural connectivity reconstructions.
CGC	http://www.cbs.umn.edu/CGC/	The *Caenorhabditis* Genetics Center, which distributes stocks of *C. elegans* strains and mutants, and many additional species as well.
NEMBASE	http://www.nematodes.org/nembase4/	The Edinburgh comparative nematode transcriptome database
Nematode.net	http://www.nematode.net/	The Washington University in St. Louis nematode genomics server
959 Nematode Genomes	http://www.nematodegenomes.org/	A collaborative wiki collating information on the many nematode genome projects underway or planned round the world and across the phylum.
SON	http://www.nematologists.org/	The US-based Society of Nematologists is the key professional organisation for nematologists worldwide.
ESN	http://www.esn-online.org/	The European Society of Nematologists
RhabditinaDB and WSRN	http://wormtails.bio.nyu.edu/Home.html	David Fitch's reference Web site including Rhabditina evolution, the Worm Systematics Resource Network, and the NYU collection of wild nematode species.

Understanding the action of genes through their mutant phenotypes is revealing, but deeper insight can be won from the molecular nature of their gene products and the details of the lesions induced by mutation. To this end, research teams started using molecular biological tools to isolate the DNA for their genes and describing the biochemical and physiological functions. This process was aided by another community project, undertaken by John Sulston, Alan Coulson, and colleagues, of the generation of a physical map of the *C. elegans* genome [9],[10]. Using a DNA fingerprinting technique, long, contiguous stretches of the chromosomes were assembled from overlapping cosmid clones. As these clones were further analysed, and the marker loci used in genetic mapping were cloned and placed on the physical map, it became ever easier to “clone your gene” from these mapped cosmids.

In the late 1980s, the nascent human genome sequencing program was looking for test beds for technologies to tackle the 3-gigabase human genome. The *C. elegans* genome had been sized at 100 megabases (Mb) [11], and the physical map of overlapping cosmids was ideally suited to the DNA sequencing technologies available. Thus the *C. elegans* genome project was born. In a few short years, the high-quality genome sequence emerging from teams in Cambridge, UK (later at the Sanger Institute), and St. Louis, United States, revolutionised the way *C. elegans* researchers did their science [12]. The publication of the near-complete sequence in 1998 [13] meant that *C. elegans* was the first animal for which the genome was known. The availability of this sequence changed the ways in which the worm could be approached experimentally, and large-scale projects examining gene expression, gene knockout phenotypes, and genetic interactions joined the roster of single-gene, focussed projects. For the human genome project, the *C. elegans* genome consortium proved that dedicated teams, using a clone-by-clone sequencing strategy and the new assembly and analysis tools they developed, could indeed tackle large genomes. Many technologies first developed and used for the *C. elegans* genome, such as fingerprint mapping of large insert clones, using yeast artificial chromosome cloning systems, and the first generation of automated gene finders, have subsequently been used widely.

## The *C. elegans* Toolkit

*C. elegans* has proved to be an excellent model research organism. It is not only easy to grow and study under the microscope, but it also is uniquely amenable to many genetic and other manipulations. Its transparency enables direct screening for defects and changes under the microscope, and technologies such as laser ablation (where individual nuclei are killed by the action of a laser directed through the objective of a microscope), and cell-specific optogenetic manipulation (where light-responsive ion channels and enzymes can be specifically induced in a single or a few cells) are key tools for cell-level investigation of neural and developmental systems. *C. elegans* can be genetically transformed by microinjection of foreign DNA, allowing transgenic analysis of gene function [14],[15]. The use of green fluorescent protein as a transgenic marker was pioneered in *C. elegans* [16]. The phenomenon of RNA interference (RNAi; where double-stranded RNA applied to the organism specifically knocks down expression of the targeted gene) was first discovered and applied in *C. elegans* [17]. *C. elegans* has proved to be uniquely susceptible to RNAi: genes can robustly be knocked down by feeding nematode cultures on *Escherichicia coli* that express double-stranded RNA from the gene of interest. The simplicity of this method means that RNAi “feeding” libraries targeting all of the genes in the genome are available for use in screening [18]. *C. elegans* can be grown in bulk liquid culture and phenotyped, sorted, and counted automatically for high-throughput screening of drugs and other treatments.

“Four-dimensional” microscopy, tracking cells in space and time through development, can be used to define the effects of developmental mutants in a tiny fraction of the time taken by Sulston and colleagues to determine the wild-type lineage [19],[20]. The small genome size and high quality of the sequence (it remains to this day the only absolutely complete animal genome) has in turn enabled all sorts of whole-genome assays. Thus, the model organism Encyclopaedia of DNA Elements (modENCODE) teams have used the full battery of next generation analysis tools (microarrays, DNA methylation analyses, deep sequencing transcriptomics, immunoprecipitation of chromatin bound to transcription factors) to define the regulation of the *C. elegans* genome through development [21],[22]. All of these global surveys, and the many thousands of single-gene and single-system analyses, are collated and cross-referenced in the openly accessible online database WormBase [23] (see Table 1 for *C. elegans* and other data resources).

The simple and accessible nervous system has permitted analysis of many aspects of nervous system development and function of wide importance, including issues such as how neural cells take on specific fates [24], how growing axons find their way and make the correct connections, and how individual neurons integrate the many inputs they experience. While *C. elegans* has very few sensory neurons (the sensory nervous system includes only 39 sensory neurons, most concentrated in the anterior amphids and labial sensillae [7]), the genome sequence surprisingly revealed over 1,200 putative G-protein-coupled transmembrane receptors likely to be involved in sensing the environment. Multiple receptors are expressed in a single neuron, and generation of appropriate responses involves intra- and inter-cellular regulation. The nervous system in *C. elegans*, as in other organisms, is closely integrated with hormonal control of physiology, including the regulation of dauer entry and exit, fat storage, body size, and longevity [25].

## *C. elegans* Is a Model Animal

The pattern of development observed in *C. elegans* is markedly different from that seen in other well-studied organisms such as fruit flies or mammals. In flies and mammals, deleting one or a few cells from an embryo usually has no effect on subsequent development: the embryos regulate to replace the structures that would have been produced by the missing cells. In *C. elegans*, however, removal of cells from the embryo is like removing tiles from a mosaic: the other cells cannot change fates to replace the missing parts. Does this mean that work on *C. elegans* is merely the study of a curiosity of little wider relevance? Mosaic development is actually common in small non-vertebrates, and may be an adaptation to the need for rapid, reliable embryogenesis [26], so *C. elegans'* developmental mechanisms are derived from regulative ancestors. Indeed, in the *C. elegans* embryo, the near-invariant pattern of the cell lineage is in fact set up by a series of complex cell–cell interactions. Importantly, this means that the processes and genetic circuits underpinning *C. elegans* development are likely to be common to all animals, and thus work on this simpler model has informed human and other research, and has had a huge impact on medical science.

The importance of *C. elegans* for the study of human biology has two facets. One is the startling finding that many of the genes in the *C. elegans* genome have close homologues in the human, and that many human disease genes are present in the worm. The simplicity of the nematode system makes it a favoured test bed for investigation of the function and interactions of these genes in biological systems affected in disease, including syndromes such as ageing and obesity. The second is the ability to ask simple, direct questions of the *C. elegans* system and thus get simple, direct answers of universal significance.

For example, Robert Horvitz, Paul Sternberg, and colleagues showed that the cell–cell and intracellular signalling pathways involved in the production of the hermaphrodite vulva (a process that takes place in the L3 and L4 stages) are common to all animals, and are also involved in embryogenesis and cancer in humans [27]. Horvitz and colleagues also were the first to define the pathway that controlled the programmed death (apoptosis) of specific cells during *C. elegans* embryogenesis [28]: this pathway is also found in humans, where it is an important regulator of cancerous growth.

As outlined above, RNAi was defined in *C. elegans*, and the phenomenon of RNAi is now known to use systems that are involved in innate immunity to viruses in humans and other organisms. Excitingly, genes encoding endogenous small RNAs, similar to the effector RNAs active in RNAi, were found in *C. elegans* through standard genetic screens investigating developmental mutants [29]. These defined the now burgeoning field of microRNAs (miRNAs), regulatory effectors critical in development and disease in humans, other animals, and plants.

Lastly, the dauer L3 is a non-ageing stage, and the genes that control entry and exit from the dauer were shown to affect the life span of *C. elegans*, even when they did not passage through dauer [30]. This ageing pathway is also effective in other animals, and analysis of Methuselah-like *C. elegans* mutants that live twice as long as wild type has implicated other deeply conserved pathways such as those of insulin signalling. These pathways are also implicated in ageing in other species, including humans.

## *C. elegans* in the Wild

In the laboratory, *C. elegans* grows and thrives in a two-dimensional world of agar plates, and copious food in the form of *E. coli*. Obviously, this is an artificial environment. *C. elegans* is often introduced as a “soil nematode” but it is very rarely isolated from soils. The reference strain used since Brenner's pioneer experiments is “N2”, established from spent mushroom compost [31], and most isolations have been from organic-rich environments such as urban compost heaps. However, while compost heaps are wilder than agar plates, they are still artificial environments constructed by humans. Where do *C. elegans* live when not living on human-concentrated rotting vegetation, or being cosseted on agar plates? A worldwide search for *C. elegans* by Marie-Anne Félix, Asher Cutter, and their colleagues has identified rotting fruits in temperate regions as a likely true wild habitat for this species [32]–[36].

This discovery has made the task of collecting wild *C. elegans* a much more reliable pursuit, but raises new questions. How does *C. elegans* get to rotting fruit? What does the species do outside the fruiting season? The answers to these questions are still being worked out, but it is likely that the dauer L3 plays a key role. The dauer is an arrested form, and dauers can be harvested from the soils around rotting fruits: it is likely that they persist in the environment until the next food source drops from the tree. Dauers of *Caenorhabditis* species are also often found attached to the outsides of insects, woodlice, and millipedes. These arthropod species probably act as transport hosts for the nematodes, carrying them from one food source to another. *C. elegans* has been isolated from temperate sites worldwide, from Australia to Africa, and Canada to Asia [32],[37]. The isolates have usually been from locations constructed by human action (e.g., compost heaps), and it is thus likely that the nematodes have been spread also by human action. Global transport of rooted plants and fruit, and wholesale transfer of soils, will also have efficiently carried *C. elegans*. As would be expected from this model, there is little global differentiation across *C. elegans* populations. Using highly variable microsatellite genetic markers, no evidence of isolation by distance was found, and small local areas contained as much genetic diversity as different continents. In this, *C. elegans* resembles the other key non-vertebrate model organism, the fruit fly *Drosophila melanogaster*. *D. melanogaster*, another lover of rotting fruit, has also been recently dispersed by human action from its origins in West Africa, and these diaspora populations show low levels of genetic distinction.

Interestingly, the “wild type” reference *C. elegans*, Brenner's N2 strain, is actually a multiple mutant, selected for growth in artificial lab conditions, and it may not be representative of most truly “wild” *C. elegans*. Wild males secrete a mucus plug over the hermaphrodite vulva during mating [38], but N2 does not plug, due to a recent loss-of-function mutation [39]. N2 nematodes range widely on the agar plates seeded with *E. coli*, leaving the bacterial lawn frequently, but most wild strains do not leave the bacterial lawns, clumping wherever the bacterial growth is thickest. This difference is due to another recent reduction-in-function mutation in N2 in a neuropeptide receptor gene [40],[41].

## Not All Nematodes Are *C. elegans*

When “traps” are laid to catch *C. elegans*, most of the nematodes that are caught are not the chosen worm. There are many bacteriovorous and fungivorous nematodes in soil and compost attracted to the rotting baits. Some of these are other *Caenorhabditis* species, such as the *C. briggsae* that Brenner initially worked on [34]. There are now about 25 known species in the genus *Caenorhabditis* [37],[42],[43] and many of these have been developed as satellite models to the main project. Using these species, it is possible to examine how the specific traits and genomic architectures of *C. elegans* came to be as they are, and thus develop predictive models of evolution. Species from other relatively closely related genera such as *Pristionchus* [44],[45] and *Oscheius* [46] have also been used as alternate models.

*Caenorhabditis* is part of a diverse radiation of terrestrial nematodes, the Rhabditina. The Rhabditina includes not only free-living species such as *C. elegans*, but also nematodes that associate with insects and other arthropods, and species that are important animal parasites. The free-living rhabditids are important members of terrestrial ecosystems, part of the ecological webs that drive soil productivity. The arthropod-associated species include those that just use their hosts for transport, and several that are pathogens or parasites of insects. Some of the insect-pathogenic nematodes have been developed as safe biocontrol agents for crop pests, and can be purchased (as arrested dauer stages) from garden stores. The Rhabditina also includes a very important group of vertebrate parasites, the Strongyloidea. Strongyloids such as the human hookworm *Necator americanus* are important determinants of human health in tropical countries [47],[48], and major efforts are underway to develop new drugs and vaccines for the devastating diseases they cause. In these efforts, *C. elegans* research plays a major role, acting as a test bed for drugs, and an archetype onto which the specific details of parasite biology can be mapped. For example, the infective stage in Strongyloids is a dauer-like L3, and discovery of drugs that prevent dauer exit, or mis-specify post-dauer development, may have important roles in community control programmes. Many agricultural animals are also susceptible to infection by a range of strongyloid species, and again *C. elegans* is used in preliminary studies for veterinary drug development.

## The Phylum Nematoda

Rhabditina is only one small part of the diversity of the phylum Nematoda. Nematodes are very diverse, not only in morphology (despite a general perception that nematodes are boring, they in fact have lots of morphological diversity), but also in size (adults from less than a millimetre to over 6 metres), life cycles (from parthenogens to complex cycles of alternating sexual strategies), and ecology (including parasites of almost all other large multicellular organisms, plant and animal). While only about 23,000 species have been described, current estimates suggest that there may be over a million nematode species on Earth [49]. Most species are members of the meiofauna that lives in marine sediments, where nematodes outnumber all other animals many fold [50]. Nathan Cobb, a pioneer nematologist, asked his readers to imagine a world where everything except the nematodes had been magically taken away: “our world would still be dimly recognizable…we should find its mountains, hills, vales, rivers, lakes, and oceans represented by a film of nematodes” [51].

**Figure 2:**
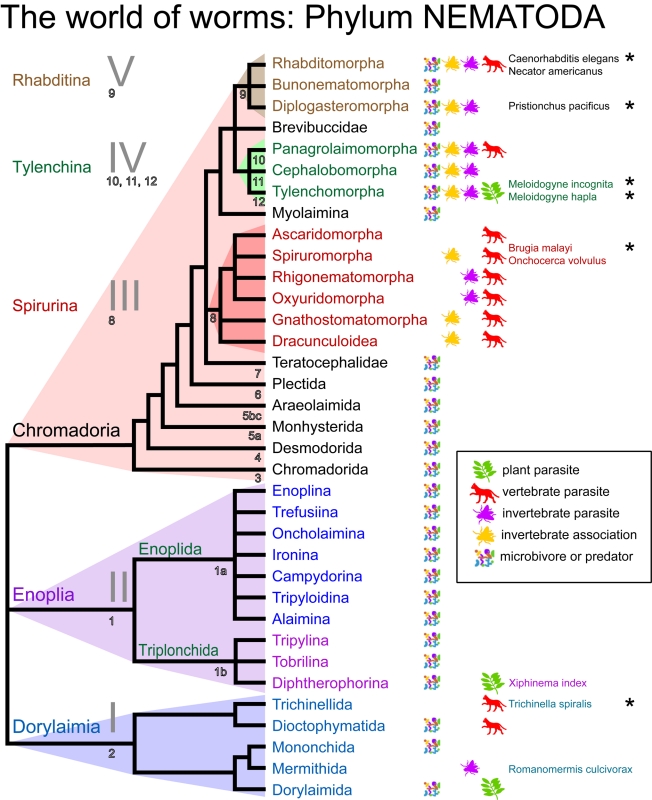
The relationships of the Nematoda. This phylogeny is based on molecular phylogenetic analyses utilising the small subunit ribosomal RNA gene. The systematic names given by De Ley and Blaxter [55],[56] are given, as is the “clade” naming convention introduced by Blaxter et al. in 1998 [52]. More recently, Helder and colleagues [53],[77] have introduced a numerical clade name scheme: this is given in outlined letters below the relevant branches. Feeding mode, and animal and plant parasitic and vector associations, are indicated by small icons, and representative species are named for some groups. Species with a sequenced genome are indicated by an asterisk.

Understanding of the phylogenetic relationships of nematodes has been changed by the use of DNA sequence data [52]–[54]. The new view of phylum Nematoda (Figure 2) [55],[56] shows three major branches, the Enoplia, Dorylaimia, and Chromadoria. *C. elegans* is placed in the Chromadoria, along with the Tylenchina (a group that includes important plant parasites, including many that devastate crops worldwide, such as *Meloidogyne incognita*, a species that can parasitise a surprisingly wide range of hosts, as well as free-living and animal parasitic species), Spirurina (which are all animal parasites, including those causing human filariases—river blindness [*Onchocerca volvulus*] and elephantiasis [*Brugia malayi*]), and other Rhabditina. In the Dorylaimia are terrestrial predatory species that play key roles in food webs, and insect and animal parasites. One of these dorylaim parasites is *Trichinella spiralis*, the trichina worm, a fascinating species that can infect many vertebrates and non-vertebrates, and causes a nasty disease in humans when diapausing larvae (the L1 stage in this case) are ingested in uncooked meats, usually pig or wild meats such as bear. The Enoplia are mainly marine, and include microbivores, predators, and a group of terrestrial herbivores (or plant parasites), the Trichodoridae. Trichodorids such as *Xiphinema index* affect their plant hosts by both feeding on the roots, and through specific transmission of devastating viruses. Parasitism of animals and plants has arisen multiple times in the Nematoda, and convergent evolution in other traits is also common [56]–[58].

One of the important results to emerge from the comparison to other nematodes is that the extreme mosaicism seen in *C. elegans* development is not found in all species [59]–[62]. Mosaic development in *C. elegans*, and related nematodes in the Chromadoria, is a derived trait. These and other comparisons are contextualising the details of the *C. elegans* project, as well as pointing out where this model nematode has followed a very idiosyncratic evolutionary path.

## Nematode Genome Projects

Research on the huge number of other nematode species does not approach that on *C. elegans* in its depth or detail, but there are especially large literatures on the human parasites and the diseases they cause. One way in which the diversity of nematodes has been approached is through comparative genomics. Initially, this was achieved through directed sequencing of the expressed genes of the target species (the transcriptome approach). Over 60 transcriptome datasets have been generated for free-living, animal-parasitic, and plant-parasitic species [63]. Furthermore, using the *C. elegans* genome project as a methodological and biological guide, teams have developed complete genome sequences for plant parasites (*M. incognita* [64] and *Meloidogyne hapla* [65]) and animal parasites (*B. malayi* [66] and *T. spiralis* [67],[68]), as well as additional free-living species (*Pristionchus pacificus* [69],[70] and additional *Caenorhabditis* species [71]). The *C. elegans* genome, at 100 Mb, is small compared to that of humans (which is 30 times bigger), but appears to be about standard for nematodes (the other sequenced species genomes range from 50 Mb to 120 Mb). The advent of new sequencing technologies has spurred a major increase in the scale of nematode genomics, and nearly a hundred genome projects are under way or planned [72]. These new genomes will reveal not only the special biology of the individual species they represent, but also expand the reach and universality of the ongoing *C. elegans* programme.

## Putting *the* Worm on the Tree of Life

Molecular data have also clarified the position of Nematoda in relation to other animals. Before the late 1990s, nematodes, along with a rag-bag of other soft-bodied, “wormy” phyla, had been placed in a group termed the Pseudocoelomata (describing the nature of the body cavity in these taxa). However, the morphological arguments supporting this superphylum were never strong, and despite the absolute certainty expressed in textbook treatments of the phylogeny of the animals, leaders in the field, such as Libby Hyman, always expressed grave doubts as to the biological reality of this grouping [73]. Analysis of ribosomal RNA sequence data from a range of nematodes, however, suggested instead a radical rearrangement of the animal part of the tree of life [74]. In this new model, which has strong support from several genes and some support from morphological data, Nematoda is part of a superphylum of moulting animals, the Ecdysozoa [74], that includes Arthropods (and thus *D. melanogaster*, the other major non-vertebrate model), Nematomorpha (horsehair *worms*), Onychophora (velvet *worms*), Tardigrada (water bears), Priapulida (penis *worms*), and other minor phyla. The rest of the “pseudocoelomates” are now placed in the Lophotrochozoa [75],[76], a group that includes Mollusca (snails and clams), Annelida (rag*worms* and earth*worms*), and Platyhelminthes (flat*worms*), amongst others.

Thus, *the* worm is only one nematode of many, and nematodes are only one sort of worm. Despite this, *C. elegans* is still a model organism *par excellence*: it is a good model nematode, and a good model animal, and a good model for the basic biology that underpins all life.

## Abbreviations

RNAi: RNA interference
