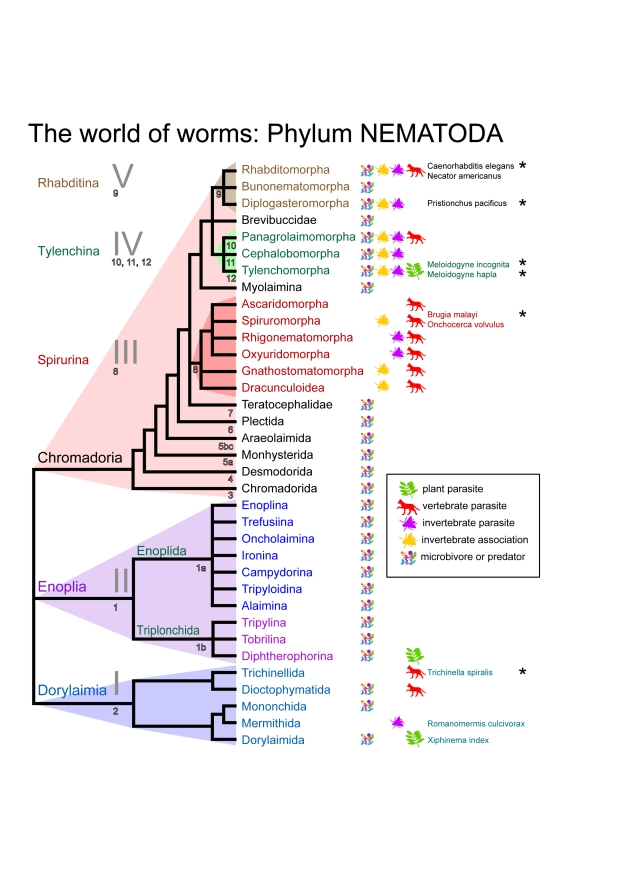# Correction: Nematodes: The Worm and Its Relatives

**DOI:** 10.1371/annotation/083d39ea-2269-4915-9297-bc6d9a9f7c58

**Published:** 2011-04-28

**Authors:** Mark Blaxter

In Figure 2, Xiphinema index is a member of the Dorylaimida, not Triplonchida. Please see the corrected Figure 2 here: 

**Figure:**